# Synthesis, Structure, and Dye Adsorption Properties of a Nickel(II) Coordination Layer Built from d-Camphorate and Bispyridyl Ligands

**DOI:** 10.3390/polym9120661

**Published:** 2017-11-30

**Authors:** Meng-Jung Tsai, Jing-Yun Wu

**Affiliations:** Department of Applied Chemistry, National Chi Nan University, Nantou 545, Taiwan; s97324905@mail1.ncnu.edu.tw

**Keywords:** chiral, dye adsorption, methyl blue, nickel

## Abstract

Reaction of NiCl_2_∙6H_2_O, d-camphoric acid (d-H_2_cam), and *N*,*N*′-bis(pyraz-2-yl)piperazine (bpzpip) in pure water at 150 °C afforded a novel nickel(II) coordination layer, [Ni_4_(d-cam)_2_(d-Hcam)_4_(bpzpip)_4_(H_2_O)_2_] (**1**), under hydro(solvo)thermal conditions. Single-crystal X-ray structure analysis reveals that **1** adopts a six-connected two-dimensional (2D) chiral layer structure with 3^6^-**hxl** topology. Dye adsorption explorations indicate that **1** readily adsorbs methyl blue (MyB) from water without destruction of crystallinity. On the contrary, methyl orange (MO) is not adsorbed at all. The pseudo-second-order kinetic model could be used to interpret the adsorption kinetics for MyB. Equilibrium isotherm studies suggest complicated adsorption processes for MyB which do not have good applicability for either the two-parameter Langmuir or Freundlich isotherm model. The saturated adsorption capacity of **1** for MyB calculated by Langmuir is 185.5 mg·g^−1^ at room temperature.

## 1. Introduction

In the past decades, coordination polymers (CPs) and metal–organic frameworks (MOFs)—virtually infinite crystalline structures of inorganic and organic building blocks, periodically repeated in one, two, or three dimensions through the connection of metal–ligand coordination bonds—have promised to be practically functional materials, especially in sorption-related fields [1,2,3]. One of the applications in CPs and MOFs that has recently received increased interest is the treatment of hazardous pollutants, such as heavy metal ions [4,5,6], organic dyes [7], pharmaceuticals, personal care products (PPCPs) [8], and so on, from contaminated water for water reuse. Colored wastewater caused by organic dyes is one of the greatest concerns due to its high visibility [9]. Moreover, organic dyes are toxic, carcinogenic, and extremely hazardous, and would therefore cause serious damage to aquatic living organisms and creature health [10,11]. Thus, organic dyes, as one of the most abundant industrial pollutants, are eco-unfriendly; they should be carefully pre-treated before discharge of dyeing effluent or contaminated water in order to reduce the impact on the environment.

Generally, there are two options reported so far to obtain clean water by using CPs and MOFs for either the adsorptive removal of organic pollutants in water [7,12] or the photocatalytic degradation of these chemicals [12,13]. As is well-known, organic dyes have been found to have high photo and chemical stability, and are difficult to degrade under natural conditions [13,14,15,16]. Thus, adsorption, with advantages of simplicity, efficiency, wide-ranging availability, and economic competitiveness, represents one of the most feasible treatment processes for dye removal from water [10,17]. The recent enthusiasm around the use of CPs and MOFs as advanced adsorbents for water purification has been documented in the past couple of years [7,11,12,18,19,20,21,22,23].

We present herein the synthesis, structure, and dye adsorption behaviors of a novel Ni(II) CP, [Ni_4_(d-cam)_2_(d-Hcam)_4_(bpzpip)_4_(H_2_O)_2_] (**1**), which was constructed from fully and partially deprotonated d-camphoric acid (d-H_2_cam) and *N*,*N*′-bis(pyraz-2-yl)piperazine (bpzpip). Dye adsorption characteristics of this Ni(II)-based crystalline material for methyl blue (MyB) and methyl orange (MO) were explored, and clearly indicate that **1** readily adsorbs MyB but not MO from water through a pseudo-second-order kinetic model. Further, it is noted that the crystallinity of the dye-loaded material of **1** is maintained during the adsorptive removal process.

## 2. Experimental Section

### 2.1. Materials and Methods

The ligand *N*,*N*′-bis(pyraz-2-yl)piperazine (bpzpip) was prepared according to the method in the literature [24]. Other chemical reagents and solvents were purchased commercially and were used as received without further purification. Infrared (IR) spectra were recorded on a PerkinElmer Frontier FT-IR spectrometer (Taipei, Taiwan) using the attenuated total reflection (ATR) technique; abbreviations: s = strong, m = medium, w = weak, vw = very weak, br = broad. X-ray powder diffraction (XRPD) patterns were obtained on a Shimadzu XRD-7000 diffractometer (Kyoto, Japan) using graphite-monochromated Cu Kα radiation (λ = 1.5406 Å), in which the X-ray tube was operated at 40 kV and 30 mA at room temperature, with a step size of 0.02° in θ and a scan speed of 1 s per step size. Thermal analyses were performed under nitrogen with a Thermo Cahn VersaTherm HS TG analyzer (Newington, NH, USA) with a heating rate of 5 °C/min. Microanalyses (C, H, N) were conducted on an Elementar Vario EL III analytical instrument (Langenselbold, Germany). UV–Vis absorption spectra were recorded on a CARy 50 Probe spectrophotometer. Brunauer–Emmett–Teller (BET) analyses were investigated with a Micromeritics TriStar II Plus analyzer (Norcross, GA, USA) using nitrogen as the adsorbate at 77 K.

### 2.2. Synthesis of [Ni_4_(d-cam)_2_(d-Hcam)_4_(bpzpip)_4_(H_2_O)_2_] *(**1**)*

A mixture of NiCl_2_∙6H_2_O (103.6 mg, 4.4 × 10^−1^ mmol), d-camphoric acid (d-H_2_cam, 30.7 mg, 1.5 × 10^−1^ mmol), bpzpip (22.4 mg, 9.2 × 10^−2^ mmol), and H_2_O (3 mL) was conducted in an acid digestion bomb at 150 °C for 48 h and then cooled to 40 °C, resulting in pale-green block-shaped crystals (40.2 mg, 1.7 × 10^−2^ mmol). The crystals were collected by filtration, washed with distilled water and ethanol, and dried at room temperature. Yield: 55% based on bpzpip. IR (ATR, cm^−1^): 3544br, 2961vw, 2924w, 2873w, 1715m, 1607m, 1585s, 1518m, 1433m, 1400m, 1361w, 1316w, 1248m, 1206w, 1139m, 1078w, 998m, 958m, 826s, 785m, 631m. Anal. Calcd for C_108_H_148_N_24_Ni_4_O_26_: C, 53.31; H, 6.13; N, 13.82. Found: C, 53.83; H, 6.03; N, 13.80.

### 2.3. X-ray Data Collection and Structure Refinement

The single-crystal X-ray diffraction data were collected at 150(2) K on a Bruker Smart APEX 2 CCD area-detector diffractometer (Billerica, MA, USA) using graphite-monochromated Mo Kα radiation (λ = 0.71073 Å). The structures were solved by direct methods with the SHELXS-97 [25] program, and refined by full-matrix least-squares methods on *F*2 using the SHELXL-2013 [26] and WINGX [27] program packages. Non-hydrogen atoms were found from the Fourier difference map and refined anisotropically, except where noted. Both of the two crystallographic distinct d-cam ligands have two statistical orientations with equal-occupancy related through a twofold rotational symmetry, which were refined isotropically because they showed NPD (non-positive definite) or had a physically unreasonable form with high ADP (atomic displacement parameter) max/min ratio upon anisotropic refinement. SADI, SIMU, and DELU commands were used to restrain the models. Carbon-bound hydrogen atoms were geometrically placed and refined in the riding model. Oxygen-bound hydrogen atoms were first located in the Fourier difference map, and then fixed at calculated positions and included in the final refinement. All hydrogen atoms were refined isotropically. CCDC 1552005 contains supplementary crystallographic data for this paper. These data can be obtained free of charge from the Cambridge Crystallographic Data via www.ccdc.cam.ac.uk/data_request/cif. Experimental details for X-ray data collection and the refinements are summarized in Table S1.

### 2.4. Dye Adsorption Measurements

The adsorption of dye from water by **1** was performed by using a batch technique at room temperature. Aqueous solutions of dye materials with desired initial concentrations were prepared by dissolving methyl orange (MO, 20 ppm) and methyl blue (MyB, 10, 20, 30, 40, 50, 100, 150, 200 ppm) in deionized water. For MyB, a calibration curve was obtained from the spectra of the standard concentrations (10–50 ppm, Figure S3). In a typical experimental setup for dye adsorption, 10 mg of as-made and inactive samples of **1** were added to an aqueous solution (4 mL) of MO with a dye concentration at 20 ppm or MyB with initial dye concentrations at 10–200 ppm. The pH of the samples was not adjusted artificially. These mixtures were stirred for about 1 minute and then allowed to sit undisturbed until measurements were taken. After adsorption for pre-determined time intervals (0, 2, 10, 20, 30, 60, 90, 120, 240, 360, 720 min), the solution was separated from the adsorbents in order to monitor the dye adsorption process by measuring the absorption spectra using a UV–Vis spectrometer (Figures S4 and S5). The dye concentration was analyzed according to the Beer–Lambert law, with absorbance at 463 nm for MO and 600 nm for MyB in the UV–Vis spectra. For MyB, if needed, a UV–Vis measurement was conducted after diluting the concentrated aqueous solution of un-adsorbed dye.

The dye removal efficiency was calculated using the formula in Equation (1):

(1) $$\mathrm{Dye}\;\mathrm{removal}\;\mathrm{efficiency}\;\left(\%\right)=\frac{C_{i}-\;C_{t}}{C_{i}}\times 100\%$$

where *C_i_* (ppm) represents the initial concentration of dye in the water and *C_t_* (ppm) represents the concentration of dye in the water at any specified time *t*.

The equilibrium adsorption amount (*Q_e_*, mg·g^−1^) was calculated using Equation (2):

(2) $$Q_{e}=\frac{\left(C_{i}-C_{e}\right)V}{m}$$

where *C_i_* and *C_e_* (ppm) represent the initial and equilibrium concentrations of dye in the water, *V* (L) represents the volume of dye solution, and *m* (g) represents the mass of the adsorbent.

## 3. Results and Discussion

### 3.1. Syntheses and Characterization

**Scheme 1:**
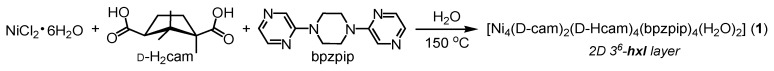
Synthesis of [Ni_4_(d-cam)_2_(d-Hcam)_4_(bpzpip)_4_(H_2_O)_2_] (**1**).

**Figure 1:**
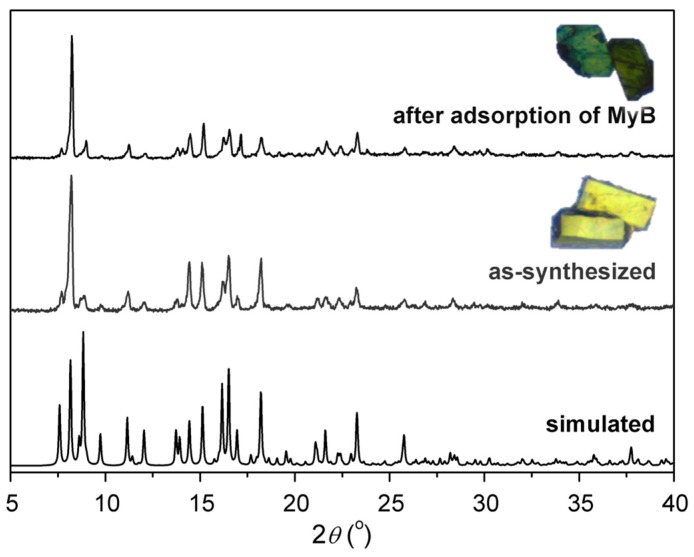
X-ray powder diffraction (XRPD) patterns of **1**. The crystal photos represent the crystals of **1** before and after adsorption of methyl blue (MyB).

Complex [Ni_4_(d-cam)_2_(d-Hcam)_4_(bpzpip)_4_(H_2_O)_2_] (**1**) as pale-green crystals was prepared by hydro(solvo)thermal reaction of NiCl_2_∙6H_2_O, d-H_2_cam, and bpzpip in pure water at 150 °C (Scheme 1). Single-crystal X-ray diffraction analysis confirmed that the crystal structure of **1** suits a two-dimensional (2D) sheet structure. The phase purity of the bulky material is characterized by X-ray powder diffraction (XRPD) measurements, which indicate that the XRPD pattern of the bulky material of **1** is in good agreement with the simulated pattern calculated from the single-crystal data (Figure 1).

### 3.2. Crystal Structure of [Ni_4_(d-cam)_2_(d-Hcam)_4_(bpzpip)_4_(H_2_O)_2_] *(**1**)*

**Figure 2:**
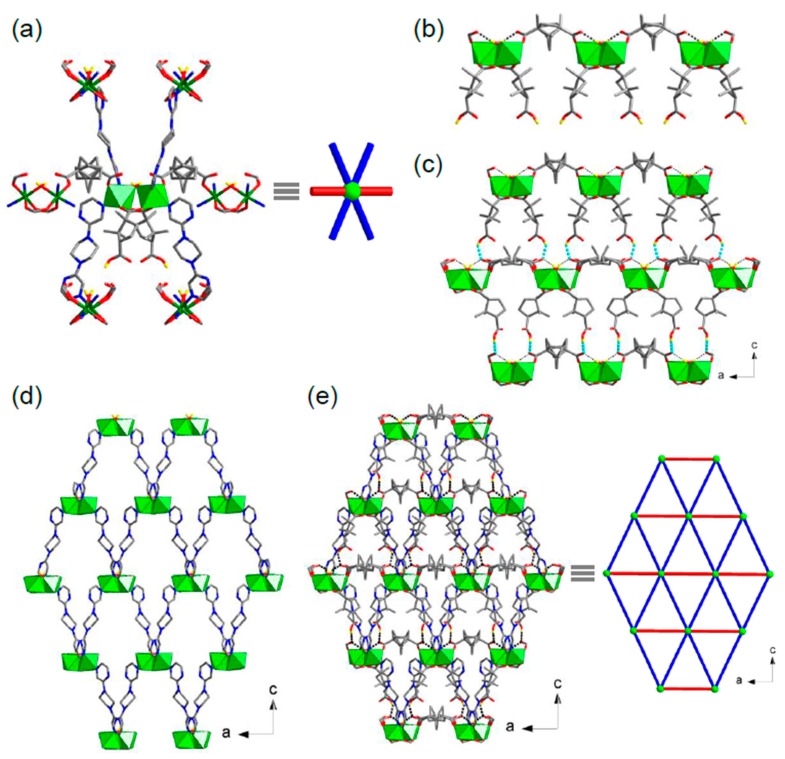
Molecular structure of **1**: (**a**) Water-bridged dinuclear cluster [Ni_2_(*μ*-H_2_O)(O_2_C)_4_N_4_] (green ball) as a topologic six-connected node (red lines: d-cam links; blue lines: bpzpip links); (**b**) The chiral nickel–camphorate coordination chain bound with d-Hcam pendants; (**c**) The 2D chiral nickel–camphorate supramolecular sheet structure supported by chain-to-chain O–H∙∙∙O hydrogen-bonding interactions (cyan dashed lines); (**d**) The achiral nickel–bpzpip (4,4)-sheet; (**e**) The overall 2D layer structure showing uninodal six-connected 3^6^-**hxl** net topology.

Complex **1** crystallizes in the orthorhombic chiral space group *P*2_1_2_1_2. From a crystallographic point of view, the asymmetric unit consists of two Ni(II) centers, two d-cam ligands (both of which are disordered over a twofold rotational symmetry), two d-Hcam ligands, two bpzpip ligands, and two bridging aqua ligands (both of which lie in a special position with a twofold rotational symmetry). All of the crystallographically distinct d-Hcam ligands have a carboxylic acid free for coordination and a *syn*,*syn*-bridging bidentate *μ*-carboxylato-*κ*^2^*O*,*O*′ group joining two Ni(II) centers (mode I in Scheme S1), whereas the distinct d-cam ligands all have two monodentate carboxylato-*κ*^1^*O* groups, each coordinating to one Ni(II) center (mode II in Scheme S1). The bpzpip ligand adopts only a *chair*-*e*,*e*-*syn* conformation and serves as a bis-monodentate ligand bridging two Ni(II) centers (Scheme S2), where *chair* means the chair conformation of the piperazine backbone, *e* means that the pyrazine-donating groups are bound at the equatorial position of the piperazine backbone, and *syn* means that the exo-nitrogen atoms of the two pyrazine rings are donated in the same side orientation. The octahedrally coordinated Ni(II) center is surrounded by four O donors from three carboxylato groups of one d-cam and two d-Hcam ligands and one aqua ligand, and two cis-positioned N donors from two bpzpip ligands. Two such Ni(II) centers are bridged by two carboxylato groups of two d-Hcam ligands and one aqua ligand to form a water-bridged dinuclear cluster [Ni_2_(*μ*-H_2_O)(O_2_C)_4_N_4_] (Figure 2a), with a Ni∙∙∙Ni separation of 3.494/3.495 Å, where moderate hydrogen-bonding interactions between the bridging aqua ligand and the monodentate carboxylato-*κ*^1^*O* groups of the d-cam ligands (O∙∙∙O, 2.34(2)–2.87(2) Å, Table S2) are formed. These corner-sharing water-bridged dinuclear [Ni_2_(*μ*-H_2_O)(O_2_C)_4_N_4_] clusters, behaving as secondary building units (SBUs), are interconnected through d-cam ligands as single-edged links to form a chiral nickel–camphorate chain, in which each SBU is further bound by two d-Hcam ligands as pendants (Figure 2b). There are significant chain-to-chain O–H∙∙∙O hydrogen-bonding interactions between the carboxylic acid of the d-Hcam pendants in one chain and the monodentate carboxylato-*κ*^1^*O* group of the d-cam ligands in the neighboring chain (2.59(2)–2.87(2) Å, Table S2), thus forming a 2D supramolecular sheet structure supported by hydrogen bonds (Figure 2c). On the other hand, interconnection of these SBUs through bpzpip ligands as single-edged links generates a 2D undulated nickel–bpzpip (4,4)-sheet structure (Figure 2d). Finally, the 2D achiral nickel–bpzpip sheets are intersected by the 1D chiral nickel–camphorate chains to result in a 2D uninodal six-connected sheet featuring the 3^6^-**hxl** topology (Figure 2e).

### 3.3. Thermogravimetric and N_2_ Adsorption Properties

The thermogravimetric (TG) trace of **1** reveals that the network is retained until it begins to decompose at temperatures approaching 155 °C (Figure S1). After a two-step decomposition of the framework ended at ca. 458 °C, the final residue of 11.6% is assigned to NiO (calcd 12.3%). The N_2_ adsorption data on thermally activated samples of **1** were collected at 77 K, which revealed a much reduced N_2_ uptake capacity (Figure S2). The estimated surface area determined by applying the BET method from the N_2_ isotherms is only 18 m^2^/g. The small value of the surface area is consistent with the crystal structure feature of the material that **1** adopts a nonporous layered network.

### 3.4. Dye Adsorption Behaviors

**Figure 3:**
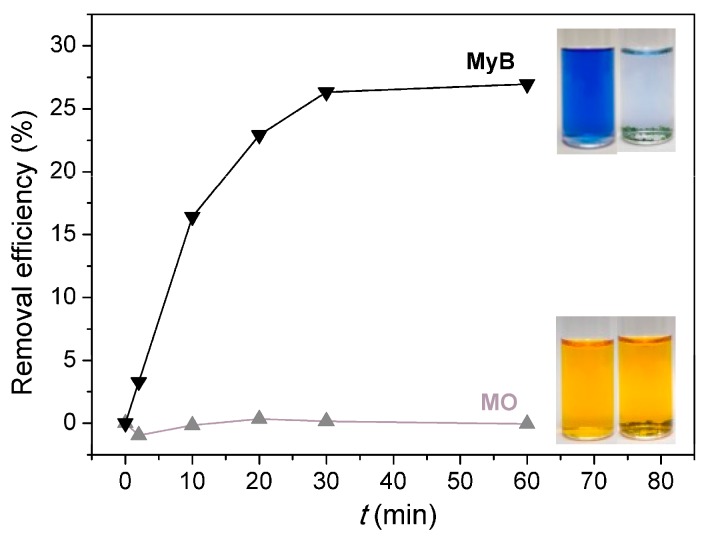
Dye removal efficiencies of **1** in a single dye solution of MyB and methyl orange (MO) in water with time. Inset photos: The color of the aqueous solutions of MyB (upper) and MO (lower) before (left) and after (right) adsorption by adsorbents for 60 min.

CPs and MOFs have recently been utilized as newly-developed adsorbents other than traditional adsorbents like activated carbon [28,29], clay minerals [30,31], zeolites [32,33], and so on [10,34,35,36,37,38,39,40,41] to adsorptively remove dyes from contaminated water. The adsorption characteristics of **1** were explored by removing harmful dye materials such as MyB and MO from water, which were monitored by time-dependent UV–Vis spectra (Figures S4 and S5). The preliminary dye adsorption studies clearly indicate that **1** does not adsorb MO from water as the orange aqueous solution of MO does not show evident color change over a period of time upon dipping the crystalline samples of **1** into the dye solution (Figure 3). This is also supported by time-dependent UV–Vis spectra. On the contrary, **1** exhibits significant dye removal behavior for MyB from water (dye removal efficiency reaches approximately 26% within 30 min), as supported by time-dependent UV–Vis spectra and obvious color change from blue to very light blue observable by the naked eye during the same process (Figure 3). Of particular note, the crystal color is also changed from pale green to deep green (Figure 1). The XRPD patterns of the dye-loaded solids collected after adsorption of MyB show profiles very similar to that observed for the fresh as-synthesized **1** (Figure 1). These facts unambiguously indicate that **1** readily adsorbs MyB from water with maintenance of structural integrity and crystallinity.

**Figure 4:**
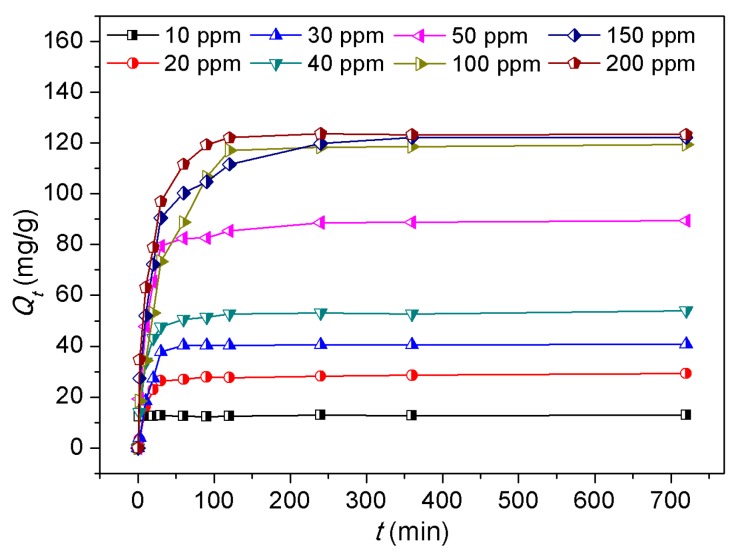
Time-dependence of adsorption profiles of MyB over adsorbent **1** at varying initial dye concentrations at room temperature.

Figure 4 depicts the adsorption amount of MyB over the adsorbent **1** as a function of time (2 min to 720 min) at room temperature, and shows the effects of different initial concentrations of MyB (10–200 ppm). Obviously, the adsorption performance of MyB over **1** increases sharply in the first 30 min and then reaches equilibrium gradually in about 2 h, suggesting a rapid adsorption process. This is explained by the fact that a vast amount of available adsorption sites is present at the beginning and, as time passes, they become saturated [42]. To achieve ultimate equilibrium, the adsorption time was prolonged up to 720 min in this study. On the other hand, the adsorption capacity of MyB on adsorbent **1** significantly increased as the initial dye concentration increased, suggesting favorable adsorption at high dye concentrations.

**Figure 5:**
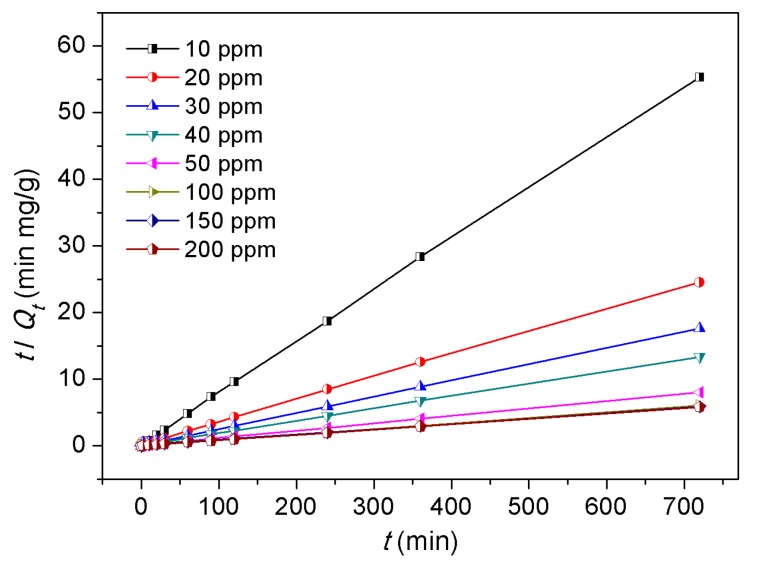
Plots of pseudo-second-order kinetics for the adsorption of MyB over adsorbent **1**.

To explore the adsorption kinetics precisely, the dynamics of adsorption were interpreted by a pseudo-second-order linear kinetic model [11,42,43], which can be expressed by Equation (3):

(3) $$\frac{t}{Q_{t}}=\frac{t}{Q_{e}}+\;\frac{1}{k_{2}Q_{e}^{2}}\;$$

where *t* (min) is adsorption time; *Q_e_* and *Q_t_* (mg·g^−1^) denote the amounts of dye adsorbed per gram of the adsorbent at equilibrium and at time *t*, respectively; and *k*_2_ (g·mg^−1^·min^−1^) is the pseudo-second-order rate constant. As shown in Figure 5, the plots of *t*/*Q_t_* against *t* for the adsorption kinetics of MyB over adsorbent **1** gave straight lines well-fitted to the experimental data with a high correlation coefficient (*R*^2^ > 0.99, Table S3), confirming a high degree of accuracy for the adsorptions in the studied concentrations. Moreover, the values of *k*_2_ decrease as the initial dye concentrations of MyB increase, which suggests not only the weaker competition of the adsorption surface sites at low concentrations [44], but also the significance of a chemisorption process being the rate-controlling step in the adsorption process for MyB [45].

**Figure 6:**
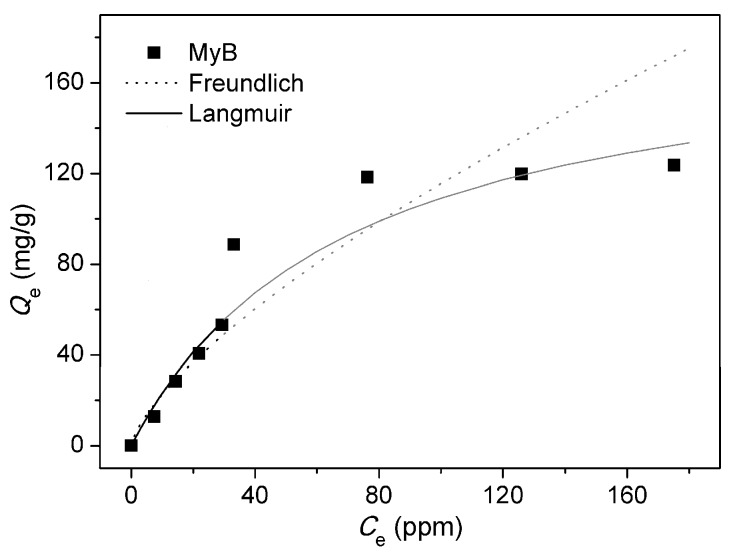
Adsorption isotherm of MyB over adsorbent **1**. Solid and dotted lines refer to the fitted adsorption isotherms for adsorbents by the non-linear Langmuir and Freundlich adsorption isotherm models, respectively.

Adsorption models are widely used to describe the adsorption progress and investigate mechanisms of adsorption. The adsorption isotherm of the MyB was obtained at initial concentrations of 10–200 ppm on **1** at room temperature by plotting the *Q_e_* as a function of *C_e_* after adsorption (Figure 6). The above-mentioned equilibrium data of **1** were firstly fitted to the Langmuir isotherm model (Figure S6a) [11,46], which is derived on an assumption that an ideal monolayer adsorption process takes place at specific equivalent sites within the homogeneous adsorbent surface. The linear form of the Langmuir isotherm model equation is given by Equation (4):

(4) $$\frac{C_{e}}{Q_{e}}=\;\frac{C_{e}}{Q_{m}}+\;\frac{1}{Q_{m}K_{L}}$$

where *C_e_* (ppm) is the equilibrium concentration of dye in water, *Q_e_* (mg·g^−1^) is the equilibrium adsorption capacity of dye, *Q_m_* (mg·g^−1^) is the theoretical maximum monolayer adsorption capacity of dye, and *K_L_* (L·mg^−1^) is the Langmuir adsorption constant related to the affinity of the binding sites. The *Q_m_*, *K_L_*, and *R*^2^ values are listed in Table 1. The calculated maximum adsorption capacity of MyB over **1** is about 185.5 mg·g^−1^, which represents a medium uptake for a wide series of adsorbents reported so far including activated charcoal (Table 2). However, this value shows **1** to be a good adsorbent to adsorb MyB from water when compared with traditional adsorbents such as activated charcoal.

Further, a dimensionless separation factor *R_L_* derived from the initial concentration of dye (*C_i_*, ppm) and Langmuir adsorption constant (*K_L_*, L·mg^−1^) can also be used to predict the favorability of an adsorption system, which is defined as Equation (5) [11,29]:

(5) $$R_{L}=\;\frac{1}{1+\;C_{i}K_{L}}$$

whereas the calculated *R_L_* values versus the initial MyB concentrations are shown in Figure S7. As observed, all the *R_L_* values ranging from 0 to 1.0 represent favorable adsorption of MyB over adsorbent **1** at the conditions being studied.

The Freundlich isotherm model [11,47], which is an empirical equation used to imply multilayer adsorption on roughly heterogeneous adsorbent surfaces caused by the presence of different functional groups on the surface and several adsorbent–adsorbate interactions, was also tested to fit the equilibrium data of MyB adsorption isotherm (Figure S6b). The linear form of the Freundlich isotherm model can be expressed by Equation (6):

(6) $$\ln Q_{e}=\ln K_{F}+\frac{1}{n}\ln\;C_{e}$$

where *Q_e_* (mg·g^−1^) is the equilibrium adsorption capacity of dye; *C_e_* (ppm) is the equilibrium concentration of dye in water; *K_F_* (mg·g^−1^) is the Freundlich adsorption constant; and 1/*n* is a measure of the surface heterogeneity, ranging between 0 and 1, and becoming more heterogeneous as its value gets closer to zero [11]. The results are given in Table 1. As a representative, the values of *n* are larger than 1, suggesting that **1** possesses a heterogeneous surface showing normal adsorption in a favorable sorption process.

Finally, it is particularly noted that applications of the two theoretical equations to fit the experimental data in this study demonstrate that correlation coefficients fitted to the Langmuir (0.8701) and Freundlich (0.8573) isotherms are very similar for adsorbent **1**, but neither give a good fit to the experimental data. This suggests that adsorbent **1** might display complicated adsorption processes for MyB for which the two-parameter isotherm models are not well applicable.

## 4. Conclusions

A novel homochiral layer structure of a Ni(II) complex, **1**, has been successfully prepared under hydro(solvo)thermal conditions via an enantioselective synthesis strategy. Dye adsorption studies clearly indicate that **1** readily adsorbs MyB, but not MO, from water with the saturated adsorption amount of 185.5 mg·g^−^^1^ through complicated adsorption processes. Adsorption kinetics for MyB could be well interpreted by the pseudo-second-order kinetic model. Thus, the new CP material appears to be a promising adsorbent candidate for efficiently and economically removing organic dyes from wastewater.

## Figures and Tables

**Table 1:** Langmuir and Freundlich isotherms for the adsorption of MyB over **1** ^a^.

Langmuir parameters			Freundlich parameters		
***Q_m_*/mg·g^−1^**	***K_L_*/L·mg^−1^**	***R*^2^**	***n***	***K_F_*/mg·g^−1^**	***R*^2^**
185.5	0.014	0.8701	1.412	4.429	0.8573

^a^ *Q_m_*, maximum adsorption capacity; *K_L_*, Langmuir adsorption constant; *n*, adsorption intensity; *K_F_*, Freundlich adsorption constant.

**Table 2:** Comparison of MyB adsorption capacities in various adsorbents.

Adsorbents	Adsorption capacity/mg·g^−^^1^	Ref.
Activated charcoal	25.25	[28]
Graphene	50	[25]
Organo-bentonite without magnetic exposure	92.96	[30]
Organo-bentonite with magnetic exposure	98.15	[30]
Magnetic chitosan-graphene oxide (MGGO)	98.52	[10]
Layered double hydroxide (LDH)-carbon dot composite	185	[35]
**1**	185.5	This work
Sodium-doped graphitic carbon nitride	200–360	[36]
β-Type strontium hydrogen phosphate (β-SrHPO_4_) nanosheet	335.68	[37]
Poly(catechol-polyethylenimine)-coated Fe_3_O_4_ nanoparticle (Fe_3_O_4_ ≅ catechol/PEI)	344.8	[38]
Graphene/*β*-cyclodextrin (GNS/β-CD) composite	580.4	[39]

## Supplementary Materials

The supplementary materials are available online at www.mdpi.com/2073-4360/9/12/661/s1.
