# Supplementary material for: Synthesis, Structure, and Dye Adsorption Properties of a Nickel(II) Coordination Layer Built from d-Camphorate and Bispyridyl Ligands
Source: Polymers (Basel). 2017 Nov 30;9(12):661. doi: 10.3390/polym9120661 (PMC6418792; doi:10.3390/polym9120661)
Supplement: Supplementary file 1 [file polymers-09-00661-s001.pdf]

Electronic Supplementary Information for

Synthesis, Structure, and Dye Adsorption Properties of a Nickel(II) Coordination Layer Built from D-Camphorate and Bispyridyl Ligands

Meng-Jung Tsai and Jing-Yun Wu*

Department of Applied Chemistry, National Chi Nan University, Nantou 545, Taiwan; s97324905@mail1.ncnu.edu.tw (M.-J.T.)

Correspondence: jyunwu@ncnu.edu.tw (J.-Y.W.)

Table S1. X-ray data collection and structure refinement for **1**

Formula of refinement model	$C_{108}H_{148}N_{24}Ni_4O_{26}$
M_w	2433.34
Crystal system	Orthorhombic
Space group	$P2_12_12$
a/Å	11.0308(4)
b/Å	21.5128(8)
c/Å	23.2148(8)
V/Å^3	5509.0(3)
Z	2
T/K	150(2)
λ/Å	0.71073
D_{calc}/g cm^{-3}	1.467
F_{000}	2568
μ /mm^{-1}	0.760
θ_{min}, θ_{max}/°	0.877, 27.242
Refl collected	113496
Unique refl (R_{int})	12291 (0.0901)
Obs refl ($I > 2\sigma(I)$)	11547
Parameters	730
R_1 [a] ($I > 2\sigma(I)$)	0.0982
wR_2 [b] ($I > 2\sigma(I)$)	0.2131
R_1 [a] (all data)	0.1017
wR_2 [b] (all data)	0.2148
GOF on F^2	1.128
Flack parameter	0.161(17)
$\Delta\rho_{max}$, $\Delta\rho_{min}$ (e Å^{-3})	1.510, –1.180

[a] $R_1 = \sum \|F_o| - |F_c\| / \sum |F_o|$. [b] $wR_2 = \{\sum[w(F_o^2 - F_c^2)^2] / \sum[w(F_o^2)^2]\}^{1/2}$.

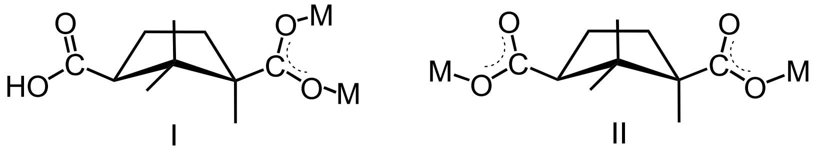

Scheme S1 Coordination modes of D-Hcam and D-cam ligands in **1**

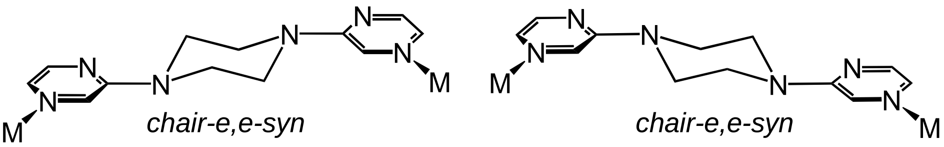

Scheme S2 Conformations of bpzpip ligand in **1**

Table S2. Hydrogen bond parameters for **1** [a]

D–H···A	*d*(D–H)/Å	*d*(H···A)/Å	*d*(D···A)/Å	∠(D–H···A)/°
O3–H101···O14#1	0.82	2.40	2.87(2)	117
O3–H101···O16	0.82	1.99	2.63(2)	134
O7–H102···O10#2	0.82	1.81	2.59(2)	160
O7–H102···O12#3	0.82	1.87	2.69(2)	170
O17–H103···O10	0.83	1.82	2.61(2)	158
O17–H103···O12#4	0.83	1.79	2.60(2)	168
O18–H104···O14	0.82	2.19	2.87(2)	140
O18–H104···O16#1	0.83	1.61	2.34(2)	146

[a] Symmetry codes: #1, $2 - x$, $1 - y$, z; #2, $1 - x$, $1 - y$, $-1 + z$; #3, x, y, $-1 + z$; #4, $1 - x$, $1 - y$, z.

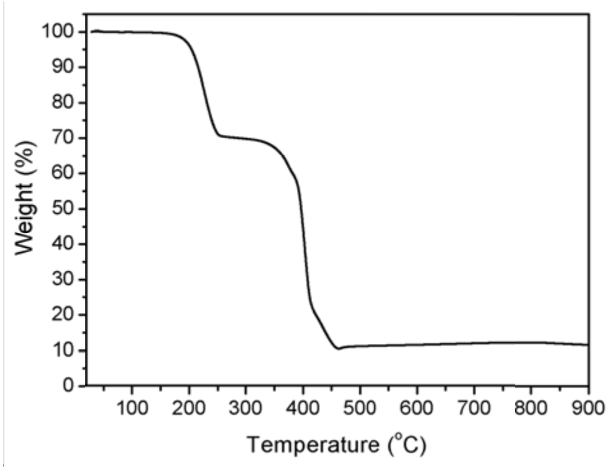

Figure S1. Thermogravimetric (TG) trace of **1**.

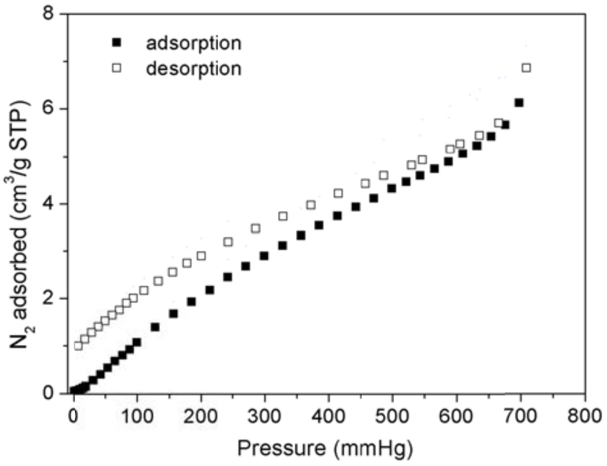

Figure S2. Nitrogen isotherms for activated **1** at 77 K. Filled markers represent adsorption, open markers denote desorption.

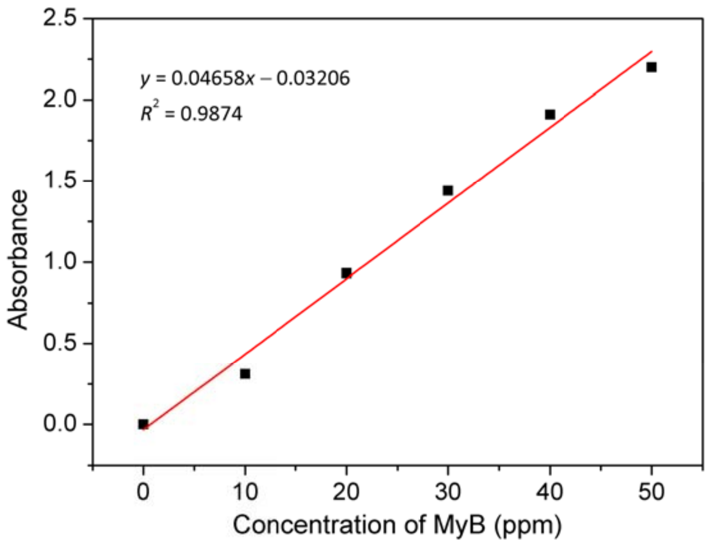

Figure S3. Calibration curve of MyB dye in water.

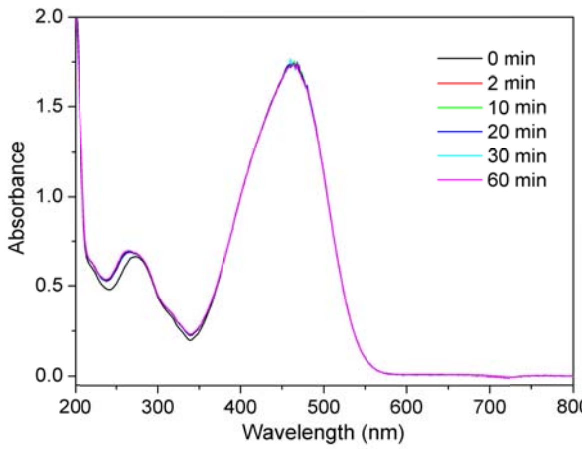

Figure S4. UV-Vis spectra of aqueous solutions of MO (20 ppm) during an adsorption test with **1**.

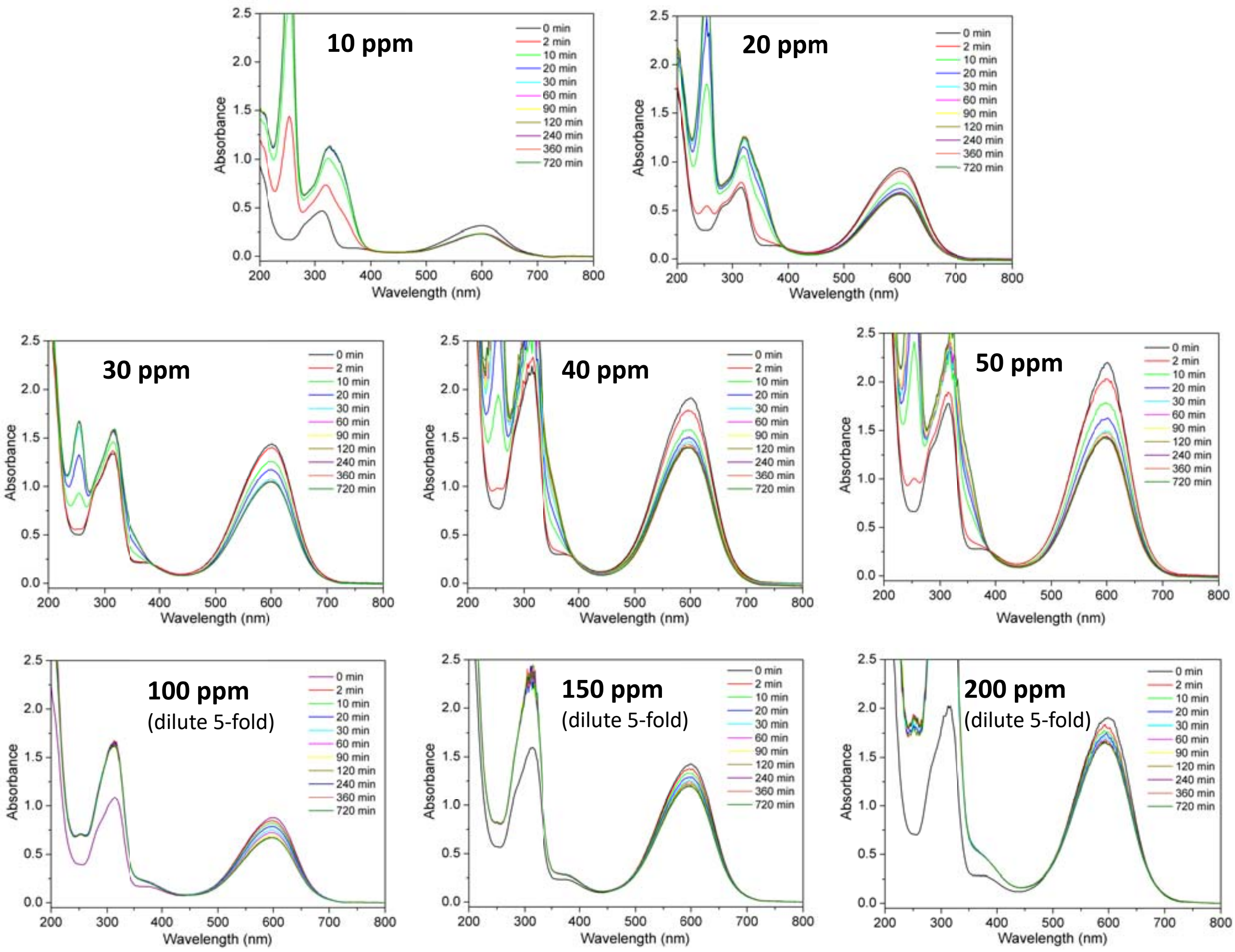

Figure S5. UV-Vis spectra of aqueous solutions of MyB during an adsorption test with **1** at varying initial dye concentrations.

Table S3. Pseudo-second-order kinetic constants for the adsorption of MyB over **1** [a]

C_i/ppm	$Q_{e,exp}$/mg g^{-1}	$Q_{e,calc}$/mg g^{-1}	k_2/g mg^{-1} min^{-1}	R^2
10	12.8	13.0	3.48×10^{-2}	0.9998
20	28.2	29.5	4.80×10^{-3}	0.9996
30	40.6	41.3	3.82×10^{-3}	0.9993
40	53.2	54.0	4.23×10^{-3}	0.9999
50	88.6	90.3	1.88×10^{-3}	0.9999
100	118.3	122.5	5.46×10^{-4}	0.9983
150	119.7	124.2	7.50×10^{-4}	0.9995
200	123.6	125.0	1.39×10^{-3}	0.9997

[a] C_i, initial dye concentration; $Q_{e,exp}$, experimental adsorption capacity; $Q_{e,calc}$, calculated adsorption capacity; k_2, pseudo-second-order kinetic constant.

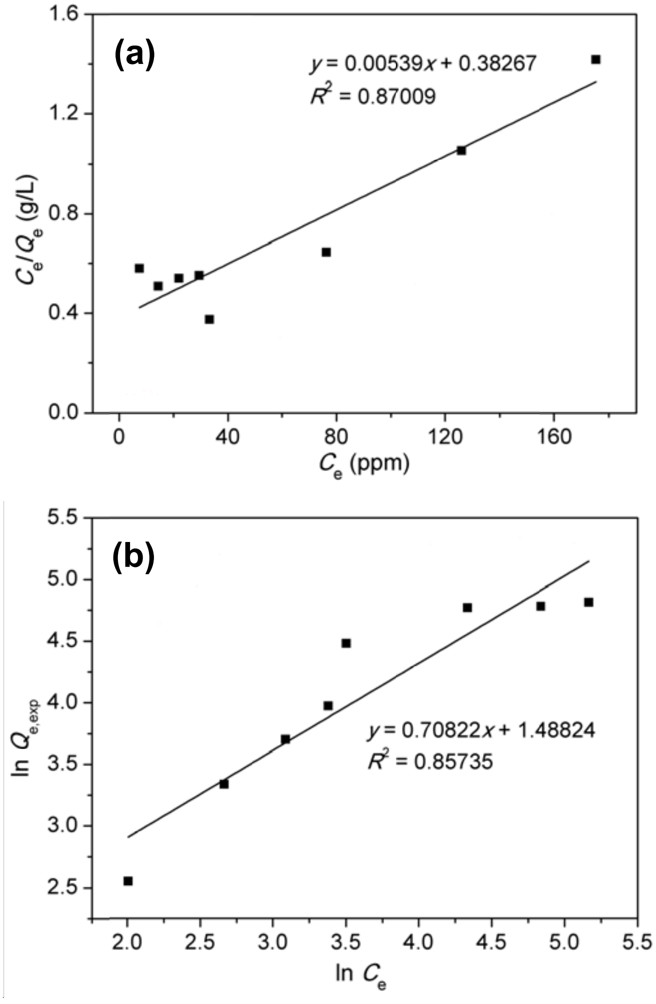

Figure S6. (a) Langmuir and (b) Freundlich plots of the isotherms for the adsorption of MyB over **1** at room temperature.

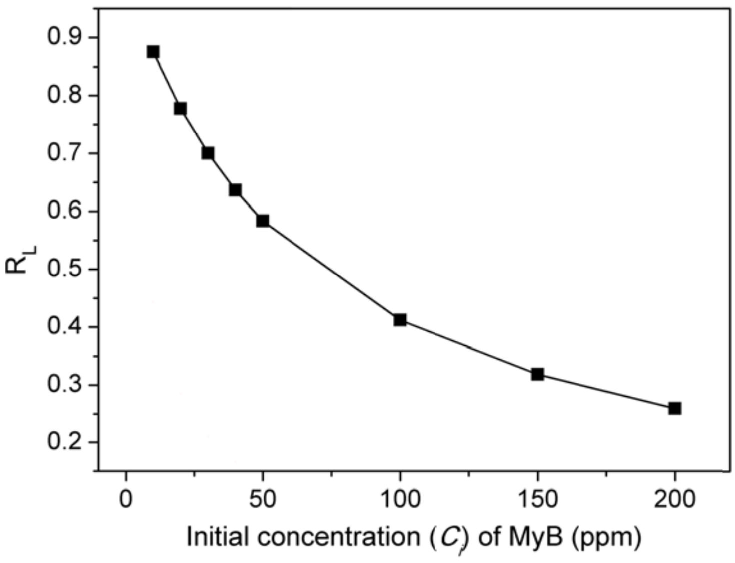

Figure S7. R_L values of the adsorbent **1** in different initial concentrations of MyB.
